# *Marinobacter subterrani*, a genetically tractable neutrophilic Fe(II)-oxidizing strain isolated from the Soudan Iron Mine

**DOI:** 10.3389/fmicb.2015.00719

**Published:** 2015-07-16

**Authors:** Benjamin M. Bonis, Jeffrey A. Gralnick

**Affiliations:** BioTechnology Institute and Department of Microbiology, University of Minnesota – Twin Cities, Saint Paul, MN, USA

**Keywords:** Fe(II)-oxidizing bacteria, genetic system, dark biosphere, deep subsurface, cultivation, characterization

## Abstract

We report the isolation, characterization, and development of a robust genetic system for a halophilic, Fe(II)-oxidizing bacterium isolated from a vertical borehole originating 714 m below the surface located in the Soudan Iron Mine in northern Minnesota, USA. Sequence analysis of the 16S rRNA gene places the isolate in the genus *Marinobacter* of the Gamma*proteobacteria*. The genome of the isolate was sequenced using a combination of short- and long-read technologies resulting in two contigs representing a 4.4 Mbp genome. Using genomic information, we used a suicide vector for targeted deletion of specific flagellin genes, resulting in a motility-deficient mutant. The motility mutant was successfully complemented by expression of the deleted genes *in trans*. Random mutagenesis using a transposon was also achieved. Capable of heterotrophic growth, this isolate represents a microaerophilic Fe(II)-oxidizing species for which a system for both directed and random mutagenesis has been established. Analysis of 16S rDNA suggests *Marinobacter* represents a major taxon in the mine, and genetic interrogation of this genus may offer insight into the structure of deep subsurface communities as well as an additional tool for analyzing nutrient and element cycling in the subsurface ecosystem.

## Introduction

Ubiquitous and abundant in marine environments, the genus *Marinobacter* contains a metabolic diversity and environmental versatility that has gained recent attention as a prospective biocatalyst for wax ester synthesis (Holtzapple and Schmidt-Dannert, 2007; Lenneman et al., 2013) and remediation of hydrocarbon contaminated environments (Strong et al., 2013; Fathepure, 2014). *Marinobacter* species become initially enriched in hydraulic fracturing effluent, suggesting an ability to thrive in harsh environments and in the deep subsurface (Cluff et al., 2014). Coupled with the prominent contribution of *Marinobacter* to geochemical cycling (Handley et al., 2009; Wang et al., 2012; Handley and Lloyd, 2013) and various ecosystem roles such as hydrocarbon degradation (Handley and Lloyd, 2013) and marine snow precipitation (Kaeppel et al., 2012), a greater understanding and control of these bacteria is necessary to fully harness and explore the processes they influence and are able to perform. Though traditionally thought to be a genus comprised exclusively of marine organisms (Handley and Lloyd, 2013), *Marinobacter* species isolated from salinous non-marine sources are challenging this definition of the genus, which includes members from wastewater (Liebgott et al., 2006), salinous soil (Martin et al., 2003), and now the deep subsurface.

The Soudan Underground Mine State Park, home of the Soudan Iron Mine, is located in the Archean Soudan Iron Formation of northern Minnesota, USA. The mine reaches a depth of 714 m below the surface, and produced high-grade hematite ore until it closed operations in 1962. In an effort to expand the depth of the mine prior to closing, core samples were drilled to locate ore-rich veins. Water from the surrounding rock, a calcium- and sodium-rich brine that reaches salinities as high as 4.2% (w/v), emerges from these boreholes. Anaerobic and containing up to 150 mM dissolved iron, the water carries with it microbes from still deeper in the earth (Edwards et al., 2006). Opposing concentration gradients of Fe(II) and oxygen form as the effluent contacts the air in the mineshaft, creating an environment conducive for aerobic microbial Fe(II) oxidation. A bacterium belonging to the genus *Marinobacter*, designated isolate JG233, was isolated from a downwardly oriented borehole located on the lowest level of the mine, approximately 714 m below the surface. A previous study conducted by Edwards et al. (2006) focusing on microbial ecology of the Soudan Iron Mine, found abundant Gamma*proteobacteria* in this level of the mine (Edwards et al., 2006). Heterotrophic cultivation estimates concentration of *Marinobacter* to be approximately 10^5^ CFU/mL from the site where JG233 was isolated (data not shown). These findings are further supported by cultivation-independent analysis where *Marinobacter* species constituted a significant fraction of 16S rDNA sequences from several sites within the mine (unpublished work). The presence of *Marinobacter* suggests the genus is well suited to survival in the high salinity and Fe(II) found in the mine, and as such is likely to influence nutrient and geochemical cycling.

The quantity of iron in the earth’s crust and its availability for electron transfer reactions enable it to significantly impact the cycling of other elements, and has been implicated in the change of state of carbon, sulfur, oxygen, nitrogen, and manganese (Ghiorse, 1984; Konhauser et al., 2011; Johnson et al., 2012). These elements play critical roles in the environment; further extending the already considerable influence of iron alone. The oxidation state of iron also influences soil structure, dissolved carbon stability (Chan et al., 2009), and enzyme activity (Baldock and Skjemstad, 2000; Bronick and Lal, 2005), affecting microbial communities and soil fertility. Additionally, microaerophilic Fe(II)-oxidizing bacteria have been implicated in the accelerated corrosion and occlusion of water-associated iron-bearing constructs, biofouling, and corroding pipes, as well as other iron-containing structures (Emerson et al., 2010; McBeth et al., 2010; Lee et al., 2013). In the environment, Fe(II)-oxidizing organisms facilitate weathering and cycling, affecting the oxidation state, bioavailability, and solubility of a variety of important elements including carbon, oxygen, nitrogen, and sulfur. Despite the importance and prevalence of these organisms, little is known regarding the biochemistry of microaerophilic Fe(II) oxidation. To our knowledge there exists no genetically tractable representative of the Fe(II)-oxidizing bacteria, and only recently has genomic work become possible. This lack of knowledge arises from two complicating factors inherent to Fe(II)-oxidizing metabolisms: the quantities of Fe(III) precipitates that result as a product of Fe(II) oxidation are inhibitory to biochemical and genetic analysis, and it is difficult to obtain sufficient biomass for analysis. These problems are coupled in current model organisms for microaerophilic Fe(II) oxidation, as growth on Fe(II) is obligatory for these organisms. For this study we set out to isolate, characterize, and describe a model organism for the study of Fe(II) oxidation capable of heterotrophic growth, and to develop a robust genetic system for this model organism. Here we describe an environmental isolate that represents a microaerophilic Fe(II)-oxidizing strain capable of heterotrophic growth, uncoupling an increase in cell density from a proportional increase in Fe(III) byproducts. Heterotrophic growth allows for the use of established molecular techniques without the complications of growth on Fe(II) normally observed in Fe(II)-oxidizing model organisms.

## Materials and Methods

### Isolation

Samples were obtained from the effluent of a descending exploratory borehole, Soudan Mine Diamond Drill Hole 942, located in the bottom (level 27) of the Soudan Underground Mine State Park in Soudan, Minnesota, USA. The effluent is an iron-rich, primarily calcium chloride brine with a circumneutral pH (6.31 ± 0.48). Strain JG233 was isolated aerobically on agar-solidified Difco Marine Broth (MB) medium (Becton Dickinson and Company, NJ, USA).

### Media and Growth Conditions

*Escherichia coli* cultures were grown at 37° C in Difco LB (Luria-Bertani) medium. When cultivated in rich medium, isolate strain JG233 was grown at 30° C in MB medium. In defined-media experiments, isolate strain JG233 was cultured at 30° C in *Mari-nobacter* Iron Medium (MIM) containing per liter: 50.0 g NaCl, 5.3 g MgCl_2_·6H_2_O, 0.75g KCl, 0.1 g MgSO_4_·6H_2_O, 50 mg K_2_HPO_4_, 1.0 g NH_4_Cl, 0.740 g CaCl_2_·2H_2_O, 0.42 g NaHCO_3_, 2.38 g HEPES (4-(hydroxyethyl)-1-piperazineethanesulfonic acid) and pH adjusted to 7.0 with 1 M HCl. LBMB (Luria-Bertani Marine Broth) medium used during conjugation contained 750 mL prepared Difco LB and 250 mL prepared Difco MB per liter. MB25RB medium used in preparation of competent cells contained per liter: 3.75 g tryptone, 1.875 g yeast extract, 1.875 g NaCl, 1mL 1N NaOH, 4.39 g MgSO_4_·6H_2_O, and 4.675 g Difco MB powder. For growth under kanamycin selection, media contained 50 μg/mL kanamycin sulfate (Fisher Scientific, MA, USA). Diaminopimelic acid was used at a concentration of 360 μM when growing *E. coli* strain WM3064. Media were supplemented with 1.5% (w/v) agar for growth on solid media.

Growth conditions were assayed aerobically in MIM at 30° C unless varied for the conditions of the assay as noted. Hetero-trophic growth of isolate strain JG233 in liquid media was monitored using optical density at 600 nm, unless the opacity of the media proved prohibitive, in which case growth was determined by counting colony forming units (CFU) on MB solid medium. Electron donors were assayed at 20 mM or 0.03% unless noted. Media for anaerobic growth were prepared by sparging MIM with N_2_ gas for 15 min in Balch tubes prior to autoclaving sterilization. Media were amended with 10 mM sodium lactate as the electron donor and 40 mM electron acceptor. Following growth in tubes containing nitrate, nitrogen speciation was determined using 0.8% N,N-dimethyl-α-naphthylamine solubilized in 5 M acetic acid and 0.6% sulfanilic acid solubilized in 5 M acetic acid, followed by the addition of powdered zinc. Salinity assays utilized 40 mM sodium lactate as the electron donor and varied concentrations of NaCl and CaCl_2_ in MIM media from 0 to 2.22 M, and 0 to 300 mM, respectively. Hydrochloric acid and sodium hydroxide were used to adjust MIM for pH assays, and contained 40 mM sodium lactate as the electron donor. Media for assaying pH tolerance contained 10 mM 3-(N-morpholino)propanesulfonic acid (MOPS) and N-cyclohexyl-2-aminoethanesulfonic acid (CHES) to extend buffering capacity. Antibiotic susceptibility was assayed using antibiotic disks AM10, N30, E15, TE30, P10, S10, and B10 (Cypress Diagnostics, Belgium) on solid LB medium.

### Fe(II) Oxidation Assay

The gel-stabilized gradient tube system from Emerson and Floyd (2005) was adapted for Fe(II) oxidation assays (Emerson and Floyd, 2005). In brief, FeS or FeCO_3_ stabilized in 1.0% agarose was deposited in the bottom of sterile Balch tubes and allowed to solidify. A low-melt agarose (SeaKem LE Agarose, Lonza, ME, USA) stabilized MIM at pH 7.0 and lacking HEPES, was gently overlaid on the Fe(II)-plugs and gassed for 1 min using 20% CO_2_ gas with a balance of N_2_. Tubes were then stoppered with butyl rubber bungs and allowed to set overnight at ambient temperature. Heterotrophically grown strain JG233 cultures were washed in defined medium lacking organic carbon before inoculation into the gradient tubes. Inoculation was performed by removing the bung from the tube, allowing atmospheric gas into the headspace, and injecting cells throughout the length of the tube. Gradient tubes were incubated statically in the dark at 30° C. During respiratory inhibition, gradient tubes of varied maturity were amended to a final concentration of 2 mM sodium azide or 20 μM N,N-dicyclohexylcarbodiimide (DCCD) solubilized in 200 proof ethanol.

### Genomic DNA Isolation and Genome Assembly

For Illumina sequencing, genomic DNA was isolated using the Wizard Genomic DNA Purification Kit (Promega, WI, USA) from an overnight culture of isolate strain JG233 grown in MB medium. Purified DNA was sequenced at the University of Minnesota Genomics Center using an Illumina platform sequencing 100 bp paired-end reads. For PacBio sequencing, pooled samples from 5 mL overnight outgrowths of isolate strain JG233 were isolated using phenol/chloroform extraction. Purified DNA was sent to the Mayo Clinic Bioinformatics Core for sequencing on a PacBio platform using size selected reads on 4 SMRT cells. Assembly was done using the Hierarchical Genome Assembly Process with 100X coverage. The genome was assembled to two contigs and polished to accuracy greater than 99.99%. Auto-annotation was performed using the RAST Annotation Server through the SEED <http://www.theseed.org/> (Aziz et al., 2008; Overbeek et al., 2014).

### Bacterial Strains and Mutant Construction

The bacterial strains, plasmids, and primers used in this study are listed in Table 1. Primers were purchased from Integrated DNA Technologies. DNA modification enzymes were purchased from New England BioLabs. DNA purification for plasmid construction was achieved using the Invitrogen Quick PCR Cleanup Kit and Quick Plasmid Miniprep Kit.

**Table 1:** **Bacterial strains, vectors, and primers**.

**Strain or vector**		**Relevant trait(s)**	**Source**
Strains			
	*Marinobacter subterrani*		
	JG233	Wild Type	This study
	JG2729	Δ*flaBG*	This study
	JG3126	Wild Type with pBBR1MCS-2	This study
	JG2756	Δ*flaBG* with pBBR1MCS-2 containing *flaBG*	This study
	JG2791	JG233 with pBBR1MCS-2 containing *gfp*mut3	This study
	*Escherichia coli*		
	UQ950	DH5α vector construction host	Saltikov and Newman (2003)
	WM3064	DAP auxotroph donor strain for conjugation	Saltikov and Newman (2003)
Vectors			
	ptpSMV3	Deletion vector, Km^r^, *sacB*	Saltikov and Newman (2003)
	ptpSMV3Δ*fla*BG	Deletion construct for *flaBG*	This study
	ptpBBR1MCS-2	Broad range cloning vector, Km^r^	Kovach et al. (1995)
	ptpBBR1MCS-2::*fla*BG	Δ*fl*a*BG* complementation vector	This study
	ptpBBR1MCS-2::*gfp*mut3	*gfp* containing vector	Laboratory stock
	ptpMiniHimar RB1	Transposon-carrying plasmid	Bouhenni et al. (2005)
Primers			
	Flagellin UF	catg**GGATCC**TTCTTCCTGTTTGGGACCGAC	This study
	Flagellin UR	catg**CTTAAG**GGCTAATGCCCCTCCAGTATC	This study
	Flagellin DF	cagt**CTTAAG**CAGTAAACTCAGAACGCCC	This study
	Flagellin DR	cagt**ACTAGT**GTGCCGGTTTCCTCGGAG	This study
	Flagellin CompF	cagt**AAGCTT**GATACTGGAGGGGCATTAGCC	This study
	Flagellin CompR	cagt**ACTAGT**GGGCGTTCTGAGTTTACTG	This study
	27F	AGA GTT TGA TCM TGG CTC AG	Wilson et al. (1990)
	M13F	GTA AAA CGA CGG CCA GT	Universal Primer List, n.d.
	ptM13R	CAG GAA ACA GCT ATG AC	Universal Primer List, n.d.

Restriction site indicated in bold.

For *flaBG* mutant construction, 1 kb regions flanking the *flaBG* genes from isolate strain JG233 were amplified by polymerase chain reaction (PCR) in two reactions, one using primers Flagellin UF/Flagellin UR, and the other with Flagellin DF/Flagellin DR (Table 1) in GoTaq Green Master Mix (Promega, WI, USA). Purified upstream and downstream amplicons were digested using either BamHI/AflII or AflII/SpeI, respectively. The vector, pSMV3 (Saltikov and Newman, 2003), was digested using BamHI/SpeI and gel purified. Fragments and vector were ligated using T4 ligase prior to transformation into *E. coli* strain UQ950.

### Preparation of Competent Cells

Chemically competent isolate strain JG233 stocks were prepared using an adapted protocol for rubidium chloride treatment. Briefly, cultures were grown to an optical density at 600 nm of 0.75 in MB25RM medium then kept on ice for the remainder of the preparation. Cultures were centrifuged and pellets resuspended in TFB1 containing per 500 mL: 1.45 g potassium acetate, 6 g rubidium chloride, 0.75g CaCl_2_·2H_2_O, 4.95 g MnCl_2_·4H_2_O, 94.5 g glycerol, and pH adjusted to 5.8 with acetic acid. Again, cells were centrifuged and pellets resuspended in TFB2 containing per 100 mL: 0.2 g MOPS, 1.1 g CaCl_2_·2H_2_O, 0.12 g rubidium chloride, and 18.9 g glycerol, and pH adjusted to 6.5 with KOH. Stocks were stored at -80° C until use. For transformation of competent isolate strain JG233, aliquots were incubated with 120 ng pBBR1MCS-2::*gfp*mut3 for 30 min on ice. Following heat shock at 45° C for 5 min, cells were incubated for an additional 2 min on ice before dilution in MB25RB. After a 60 min recovery at 30° C, cells were plated to MB plates and MB plates supplemented with kanamycin.

### Conjugation

*Escherichia coli* donor strain WM3064 was used to conjugate plasmids into isolate strain JG233. Cultures of donor and recipient were grown in LB amended with kanamycin and MB, respectively, and then diluted to an optical density at 600 nm of 1.0 with LBMB. Donor cultures were washed once with LBMB to remove kanamycin, pelleted, and decanted. After recipient cells were heat shocked at 45° C for 5 min, 1 mL was removed and used to resuspend the donor pellets. Cultures were pelleted, resuspended in 100 μL LBMB, and spotted onto LBMB plates. Following an 18 h incubation at 30° C, spots were resuspended in LBMB and spread to MB plates devoid of diaminopimelic acid and amended with kanamycin for selection against the donor strain and for the selection of merodiploid mutants, respectively. Kanamycin resistant colonies were verified by PCR using insertion-specific primers. Selection on MB plates amended with 100 mM sucrose yielded kanamycin-sensitive colonies. Mutants were confirmed via Sanger sequencing of the 16S rRNA gene using 27F and genomic DNA using Flagellin UF and Flagellin DR primers to assure no contamination had occurred.

### Phylogeny

Phylogenetic analysis was done using the two identical complete 16S rRNA genes of isolate strain JG233 obtained from the genome assembly. All sequences were size-adjusted, aligned, and a tree was constructed using the MEGA6.06 software package (Tamura et al., 2013) with the Neighbor-Joining statistical method with a Jukes-Cantor substitution model and 1000 bootstrap replications (Jukes and Cantor, 1969). Analysis of *gyrB* was conducted similarly to the 16S rRNA genes using the MEGA6.06 software package (Tamura et al., 2013) utilizing the Maximum Likelihood method and the Tamura-Nei model with 1000 bootstrap replications (Tamura and Nei, 1993). Genome comparison was conducted using the Average Nucleotide Identity calculator developed by the Konstantinidis Lab, Georgia Institute of Technology, GA, USA (Goris et al., 2007).

## Results

### Growth Conditions

Growth in MIM was supported by acetate, citrate, fumarate, fructose, glucose, glutamine, glycerol, lactate, n-hexane, n-tetradecane, succinate, and sucrose. No growth was observed in defined media containing arabinose, galactose, glucosamine, lactose, malate, or *N*-acetylglucosamine as the sole electron donor. Anaerobic oxidation of lactate was coupled to the reduction of nitrate to nitrite. Anaerobic growth was not supported by nitrite, sulfate, fumarate, DMSO, or TMAO, and no growth was observed in controls lacking a terminal electron acceptor. Isolate strain JG233 grew at temperatures between 2 and 37° C, and between pH 5 and 9, with optimum growth rates at 37° C and pH 6. Strain JG233 is capable of growth in NaCl concentrations of 0.5–13% with an optimum at 7%. Isolate strain JG233 displayed zones of clearing around diffusion disks containing ampicillin, neomycin, erythromycin, tetracycline, penicillin, and streptomycin and no apparent growth defect from bacitracin.

### Genome Properties

The draft genome of strain JG233 consists of two contigs of a single amplicon, encompassing 4,453,613 bp, 4,155 possible coding regions, and a G+C content of 58.9%. The assembly finished had an N_50_ of 3,410,286 bp and a length cutoff of 15,849 bp. Despite read lengths up to 40 kb, the assembly was unable to resolve two repetitive regions containing phage sequences of high similarity. Analysis of the SEED metabolic reconstruction using the KEGG database supports strain JG233 prototrophic for amino acid biosynthesis, which is empirically supported by growth in defined media. Analysis of the genome also suggests complete glycolysis, Entner-Doudoroff, pentose phosphate, and tricarboxylic acid cycle pathways. Carbon source utilization is supported by the genomic annotation. Based on genomic analysis, strain JG233 should be capable of fermentation and be able to utilize nitrite as a terminal electron acceptor, though cultivation-based efforts have failed to demonstrate either.

In an effort to identify gene products that may be involved in Fe(II) oxidation, sequences of proteins thought to be involved with Fe(II) oxidation in other organisms were queried against the strain JG233 translated genome. Though a putative protein with 32% identity to the CycA1 of the acidophilic Fe(II)-oxidizer *Acidothiobacillus ferrooxidans* was identified, the lack of genes encoding Cyc2 or Rusticyanin fails to suggest that a similar Fe(II) oxidation system is employed by strain JG233. Strain JG233 lacks homologs to proposed photosynthetic Fe(II) oxidation systems PioABC (Jiao and Newman, 2007) and FoxEYZ (Croal et al., 2007) from *Rhodopseudomonas palustris* TIE-1 and *Rhodobacter ferrooxidans* SW-2, respectively, as well as the proposed MtoAB and CymA_ES–1_ (Liu et al., 2012) of the neutrophilic Fe(II)-oxidizer *Sideroxydans lithotrophicus* ES-1. PioAB and MtoAB share high sequence identity with MtrAB of the Fe(III)-reducing system of *Shewanella oneidensis* MR-1. Homology between these systems suggests other genes involved with Fe(III) reduction may share orthologs in Fe(II)-oxidizing organisms, and the genome of JG233 was queried and found to be lacking homologs to MtrABCDEF, DmsEF, OmcA, and the translated products of SO_4359 and SO_4360 of *Shewanella oneidensis* MR-1. The relevance of *c*-type cytochromes in electron transfer systems was also exploited to identify candidate genes for Fe(II) oxidation. The translated genome of strain JG233 was searched for proteins containing the characteristic *c*-type cytochrome binding CXXCH motif (Kranz et al., 2009), yielding 33 possible *c*-type cytochromes. Of these, four contained two heme *c*-binding motifs, and none more than two.

### Phylogeny

**FIGURE 1:**
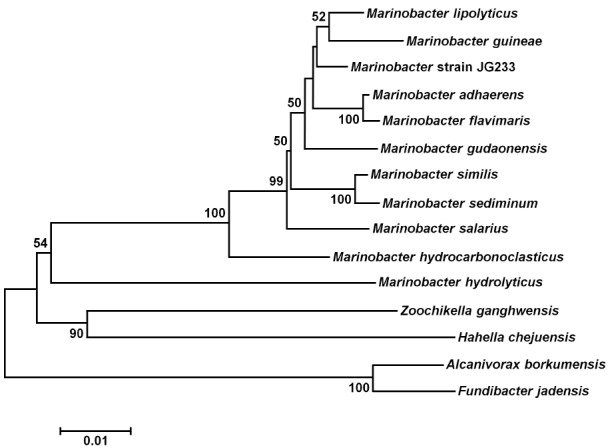
**Maximum Likelihood phylogenetic tree of the 16S rRNA gene placing *Marinobacter subterrani* JG233 in the genus *Marinobacter*.** Evolutionary relatedness was inferred using the Neighbor-Joining method (Saitou and Nei, 1987) based on the Jukes-Cantor method (Jukes and Cantor, 1969). Branch lengths are measured in the number of substitutions per site. Sequences were obtained from the National Center for Biotechnology Information, and were trimmed to as to attain a total of 1417 positions in the final dataset representing similar regions of the 16S rRNA gene. Evolutionary analyses were conducted in MEGA6 (Tamura et al., 2013). Accession numbers for included species are as follows: *Marinobacter adhaerens* (NR_074765), *Marinobacter similis* (KJ547704)*, Marinobacter gudaonensis* (NR_043796), *Marinobacter salarius* (KJ547705), *Marinobacter lypolyticus* (NR_025671), *Marinobacter flavimaris* (NR_025799), *Marinobacter sediminum* (NR_029028)*, Marinobacter guineae* (NR_042618), *Marinobacter hydrocarbonoclasticus* (NR_074619), *Zooshikella ganghwensis* (AY130994)*, Microbulbifer hydrolyticus* (AJ608704), *Alcanivorax borkumensis* (Y12579), *Fundibacter jadensis* (AJ001150), and *Hahella chejuensis* (AF195410).

**FIGURE 2:**
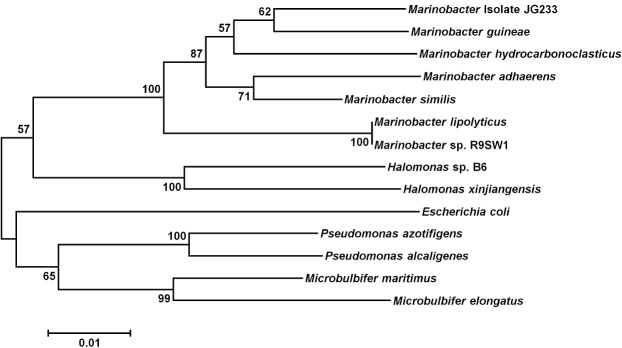
**Maximum Likelihood phylogenetic tree of the *gyrB gene*. Evolutionary relatedness was inferred using the Maximum Likelihood method based on the Tamura-Nei model (Tamura and Nei, 1993).** Branch lengths are measured in the number of substitutions per site. Sequences were obtained from the National Center for Biotechnology Information, and were trimmed so as to attain a total of 851 positions in the final dataset representing similar regions of the *gyrB* as constrained by sequence available from the National Center for Biotechnology Information. Evolutionary analyses were conducted in MEGA6 (Tamura et al., 2013). Accession numbers for included species are as follows: *Marinobacter adhaerens* (KF811467), *Marinobacter salarius* (KJ547705), *Marinobacter similis* (CP007151), *Marinobacter guinea* (KJ467768), *Marinobacter* sp. R9SW1 (KF811464), *Marinobacter hydrocarbonoclasticus* (KF811470), *Halomonas sp. B6* (KC935335), *Halomonas xinjiangensis* (KC967623), *Pseudomonas azotifigens* (KM103930), *Pseudomonas alcaligenes* (AB039388), *Microbulbifer maritimus* (AB243198), *Microbulbifer elongatus* (AB243199), *Escherichia coli* (AB083821).

Analysis of the 16S rRNA gene of isolate JG233 places it in the genus *Marinobacter*, however, members of the genus *Marinobacter* often share a greater than an 97% identity of the 16S rRNA gene (Figure 1). Few of the *Marinobacter* have additional publicly available sequences, making further sequence-based analysis difficult. Taxonomic classification of isolate JG233 was refined though comparison of gene *gyrB*, average nucleotide identity, and phenotypic properties. The *gyrB* gene is commonly used as an indicator of phylogenetic relatedness, as it contains sufficient length to tolerate subtle mutations that accumulate during speciation, but remains a highly conserved supplement or alternative to classification based on the 16S rRNA genes (Rajendhran and Gunasekaran, 2011). Establishment of strain JG233 as a distinct species from *M. adhaerens*, *M. salarius*, *M. similis*, and *M. flavimaris* is supported by comparison of *gyrB*, which clusters isolate JG233 solely, but distinctly, with *M. guineae* (Figure 2). Genomes publicly available for species with an identity higher than 97% to the 16S rRNA gene of isolate JG233 include *M. adhaerens*, *M. salarius*, *M. similis*, and *M. lypoliticus*. Two-way average nucleotide identity comparison to the strain JG233 genome yield values of 84.73, 79.51, 80.15, and 77.23% respectively. These values fall conservatively outside the 95% threshold put forth by Goris et al. (2007) for speciation.

### Genetic System

**FIGURE 3:**
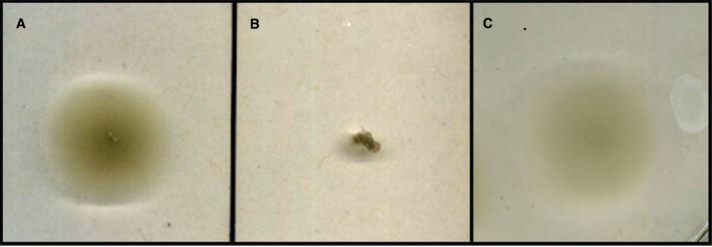
**Swimming motility assay.** Comparison of wild type *Marinobacter* strain JG233 **(A)** with Δ*flaBG* **(B)** and the Δ*flaBG* mutant complemented with pBBR1-MCS2:: *flaBG* **(C)** on 0.3% agar Marine Broth plates.

Conjugation of the pMiniHimar RB1 transposon vector into wild-type isolate strain JG233 resulted in kanamycin-resistant colonies. Presence of the transposon was confirmed by sequencing purified genomic DNA using transposon specific primers (data not shown). Direct transformation of isolate strain JG233 with plasmid pBBR1MCS-2::*gfp*mut3 was achieved by chemically induced competency with an efficiency of 1.36 × 10^4^ CFU/μg plasmid DNA. Directed deletion of the chromosomal region containing flagellin encoding genes *flaG* and three copies of *flaB* was conducted via conjugation of the suicide vector pSMV3Δ*flaBG* into wild type isolate strain JG233, followed by sucrose counter selection. Flagellin deletion mutants were confirmed by Sanger sequencing and a lack of motility in 0.3% agar MB plates (Figure 3). Motility was recovered upon conjugation of the pBBR1MCS-2::*flaBG* complementation construct into motility-deficient mutants (Figure 3C), and motility was not observed by the pBBR1MCS-2 empty-vector controls (data not shown).

### Fe(II) Oxidation

**FIGURE 4:**
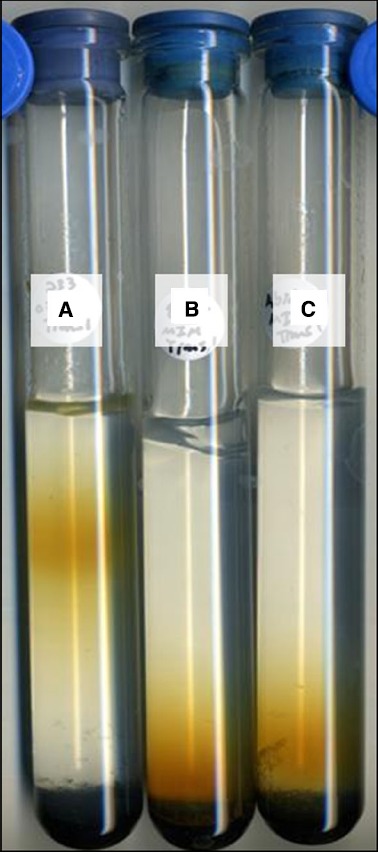
**Fe(II) oxidation by *Marinobacter strain JG233 in gradient tubes*.** Representative set of tubes (*n* = 3) demonstrating Fe(II) oxidation by *Marinobacter* strain JG233 **(A)** compared to *Escherichia coli* strain UQ950 **(B)** and an abiotic control **(C)** in tubes containing FeS as the Fe(II) source and MIM +0.15% low melt agarose as the medium overlay.

**FIGURE 5:**
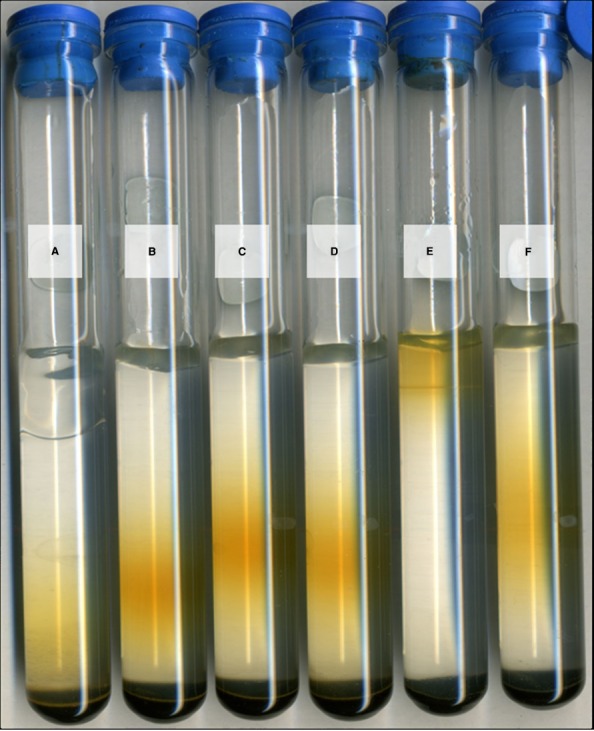
**Fe(II) oxidation under respiratory inhibition.** Representative set of *Marinobacter* strain JG233 in tubes containing FeS as the Fe(II) source and MIM +0.15% low melt agarose as the medium overlay. Results were consistent regardless of incubation time prior to respiratory inhibition, as long as inhibitor was added prior to rust-colored band formation. Tubes **(A)** and **(B)** contain sodium azide to 2 mM both abiotic and with *Marinobacter* strain JG233, respectively. N,N-dicyclohexylcarbodiimide solubilized in 200 proof ethanol was added to a final concentration of 20 μM to an abiotic control **(C)** and a tube with *Marinobacter* strain JG233 **(D)**. The tubes containing respiratory inhibitors exhibited rust-colored bands dissimilarly to uninhibited *Marinobacter* strain JG233 **(E)** and are located in similar positions as the abiotic control **(F)**, indicating Fe(II) oxidation by *Marinobacter* strain JG233 required actively metabolizing cells.

Isolate strain JG233 was tested for Fe(II) oxidation in gradient tubes and positively demonstrated Fe(II) oxidation with either FeS (Figure 4) or FeCO_3_ (results not shown) as the Fe(II) source as compared to abiotic and *E. coli* UQ950 controls. Growth was not observed in gradient tubes when compared to controls lacking Fe(II). Use of respiratory inhibitors sodium azide and N,N-dicyclohexylcarbodiimide abolished the distinct band of Fe(II)-oxides associated with microbially facilitated Fe(II) oxidation, resulting in banding consistent with abiotic Fe(II) oxidation (Figure 5). Ethanol was found to have no effect on the system in the volumes used to solubilize N,N-dicyclohexylcarbodiimide (results not shown). Scanning electron microscopy suggests cells do not form biogenically templated Fe(III) structures such as stalks or sheaths (results not shown).

## Discussion

Prior to the cessation of mining operations in 1962, expansion of the Soudan Iron Mine was actively underway and involved the drilling of cores to survey the surrounding rock. Shortly after, anaerobic and Fe(II)-rich water started welling from the deeper of these boreholes. The effluent of these boreholes is ideal for microaerophilic Fe(II)-oxidizing bacteria as oxygen diffusion forms opposing Fe(II) and oxygen gradients. Isolated from one such vertical borehole originating 714 m below the surface and almost 1,600 km from an ocean, isolate JG233 attests to the ubiquity of the genus *Marinobacter*, a genus thought to primarily consist of marine organisms. *Marinobacter* constitutes a dominant genus in the mine, with representation in some mine samples greater than 70% based on 16S rDNA sequencing (unpublished data).

### Description of *Marinobacter subterrani* sp. nov

*Marinobacter subterrani* (suhb.tuh.reyn.ahy. Genitive noun *subterrani* meaning “of the subterrane,” referring to the ecosystem from which the strain was isolated). Cells are motile rods approximately 1.8–2.0 μm in length and 0.4 μm wide under Fe(II)-oxidizing conditions and 1.5 μm in length and 0.7 μm wide when grown under heterotrophic conditions. Colonies on MB agar are smooth, convex, circular, and have a diameter of approximately 1–2 mm after 48 h of growth at 30° C. Growth occurs from pH 5–9 with an optimum around 6. Strain is moderately halophilic and capable of growth between NaCl concentrations of 0.5–13% (w/v) with an optimum at 7% (w/v). Interestingly, calcium is required for growth, with an optimum concentration of 0.54% (w/v) and a maximum tolerated concentration of 3.5% (w/v), though strontium can substitute for calcium to support growth. Divalent calcium is a major cation in the mine, reaching concentrations of 1.8% (w/v) at the sampling site.

The similarity to mine conditions of *Marinobacter subterrani* JG233 optima for salinity and pH, tolerance to calcium, and growth at 11° C, coupled with the prevalence of *Marinobacter* in the mine, suggest *M. subterrani* is not only able to propagate but is well acclimated to the conditions from which it was isolated and has not recently been introduced to this environment. Despite significant 16S rDNA similarity to other members of the *Marinobacter*; average nucleotide identity, comparison of *gyr*B, distinct environmental niche, and physiological differences support the establishment of *M. subterrani* as a distinct species (Table 2).

**Table 2:** ***Marinobacter subterrani* JG233 and defining species attributes of related species**.

**Characteristics**	**1**	**2**	**3**	**4**	**5**	**6**	**7**
DNA G+C Content (mol%)	52.7%	57.1%	58.9%	56.9	58%	57.6	57.1
Salinity range (%^a^ of Na^+^)	0.5–20.5	1–15	1–13	0.5–20	20	0.5–20	0.5–20
Temperature range (C^o^)	10–45	4–42	2–37	4–45	4–45	4–40	4–40
pH Range	6–9.5	5–9.5	5–9	5.5–10	5.5–8.0	6–9	6–9
Plasmids	2 (0.2 Mb)		0	2		0	0
Utilization of:							
Acetate	+		+			–	+
Citrate	+	–	+	–		–	–
Fumarate	+		+				
Lactate			+	+		+	+
Malate			–	+			
Succinate	+		+			–	+
Arabinose	–		–	–	–	–	–
Fructose	–	+	+		+	–	+
Glucose	–	+	+	–	–	–	–
Glycerol	–	+	+	–	–	–	+
Lactose	–		–		–	–	–
Mannitol		–		–	–	–	–
NAG	–	+	–	–		–	–
Starch		+					
Sucrose	–		+	–	–	–	–
Hydrocarbons	+		+				
Reduction of nitrate	+	+	+	–	+	+	–
Reduction of nitrite	+	+	–		–	–	–
Fermentative		+	–				

^a^in weight per volume.

NAG, N-acetyl-glucosamine.

Spaces left blank were not assayed in the original characterization paper of the species. 1, M. hydrocarbonoclasticus (Gauthier et al., 1992); 2, M. guinea (Montes et al., 2008); 3, Marinobacter subterrani JG233; 4, M. adhaerens (Kaeppel et al., 2012); 5, M. flavimaris (Yoon et al., 2004); 6, M. similis (Hooi et al., 2014); 7, M. salarius (Hooi et al., 2014).

The genome of *M. subterrani* does not contain components of any known carbon fixation pathways, and lithoautotrophic growth is neither expected nor observed (data not shown). The lack of autotrophic growth with Fe(II) as an electron donor presents a challenge to determine the role of Fe(II) oxidation by this organism, as growth in the absence of alternative electron donors normally implicates the electrons from Fe(II) oxidation are used electrogenically. The inhibition of facilitated Fe(II) oxidation by respiratory inhibitors supports Fe(III)-precipitation by *M. subterrani* requires actively metabolizing cells, and thus is not thought to occur due to an unmediated process, such as production of acidic exopolysaccharides (Chan et al., 2009), which would persist regardless of viability. Regardless of mechanism or function of Fe(II) oxidation by *M. subterrani*, the prevalence of this organism, and the abundance of *Marinobacter* species isolated from cathodic enrichments from other oxic-anoxic interface environments (Rowe et al., 2015; Wang et al., 2015), imply this genus plays a major role in the communities that dominate Fe(II)-oxidizing environments.

Current models for the study of microaerophilic Fe(II) oxidation lack the necessary genetic tools to properly interrogate the possible mechanisms responsible for Fe(II) oxidation. This lack of a robust genetic system stems from the obligate nature of these model organisms for ferrotrophy, inherently coupling the growth of the organism to be studied to the production of Fe(III) oxides. The ability to grow *M. subterrani* under heterotrophic conditions enables the use of well established methods for genetic manipulation separately from phenotypic observations when incubated with Fe(II). The establishment of a robust genetic system in *M. subterrani* has application beyond the study of Fe(II) oxidation. Phylogenetic analysis using 16S rRNA gene would suggest inclusion of *M. subterrani* in the genus *Marinobacter* of the Gamma*proteobacteria*. *Marinobacter* are well regarded as a metabolically diverse clade, containing representatives capable of many biotechnologically and industrially relevant biochemical processes. The development of a genetic system for *M. subterrani* JG233 expands upon the genetic system for *M. adhaerens* reported by Sonnenschein et al. (2011) for the analysis and development of the genus *Marinobacter* and establishes a crucial genetic system for the study of microaerophilic Fe(II)-oxidizing species (Sonnenschein et al., 2011). The genetic tools outlined in this study allow methods previously unavailable in a microaerophilic Fe(II)-oxidizing strain to be utilized. Transposon mutagenesis, markerless deletion, and complementation via expression from a plasmid enable the interrogation of the function of specific gene products, as well as the use of hypothesis generating experiments.

The isolation, description, and development of a genetic system in *M. subterrani* JG233 establishes a foundation for the genetic study of dominant organisms contributing to Fe(II) oxidation in the subsurface. As *Marinobacter* species are increasingly found in diverse environments, including hydraulic fracturing effluent, deep marine sediments, and beneath the iron ranges of Minnesota, a greater understanding of these microbes will shed light on survival and metabolism in the dark biosphere. Future studies should focus on the mechanism of the demonstrated Fe(II) oxidation and the contribution and mode of *M. subterrani* metabolism to the microbial community structure and its functioning in the native environment.

### Conflict of Interest Statement

The authors declare that the research was conducted in the absence of any commercial or financial relationships that could be construed as a potential conflict of interest.
